# Time-of-day of infection: impact on liver stage malaria parasites in untreated and drug-treated hosts

**DOI:** 10.1186/s13071-025-06986-7

**Published:** 2025-08-06

**Authors:** Petra Schneider, Aidan J. O’Donnell, Alejandra Herbert-Mainero, Sarah E. Reece

**Affiliations:** 1 Institute of Ecology and Evolution, University of Edinburgh, Edinburgh, UK; 2 Department of Human Genetics, University of Utah, Salt Lake City, USA

**Keywords:** *Plasmodium berghei*, Circadian rhythm, Active phase, Drug treatment, Liver stage, Transmission

## Abstract

**Background:**

Circadian clocks are thought to have evolved owing to the benefits of anticipating daily environmental rhythms. Daily environmental rhythms that impact on fitness include interactions between organisms, such as host–pathogen interactions. For example, host susceptibility to infection for taxonomically diverse hosts and pathogens varies across the circadian cycle. We previously revealed that mosquito vectors are less susceptible to malaria (*Plasmodium*) infection during their active phase (night time), and here we test whether a similar pattern occurs for infection of the mammalian host.

**Methods:**

We used *Plasmodium berghei*-infected *Anopheles stephensi* mosquitoes to infect mice during their rest or active phase, both in untreated and pyrimethamine-treated mice. We assessed the parasites’ success in establishing at the first site of replication (in the liver) by quantifying parasite burdens using quantitative PCR (qPCR), adjusted for sporozoite inocula. By independently manipulating the photoschedules of vectors and hosts, we standardise the time-of-day for parasites and mosquitoes used to initiate infections, and thus, directly test the impact of host time-of-day on the parasites’ ability to establish an infection.

**Results:**

The three experiments we conducted showed that pyrimethamine treatment reduced parasite liver burdens, but not in a biologically significant manner dependent on host time-of-day (active/rest phase). Furthermore, host time-of-day did not affect parasite liver burdens in untreated hosts.

**Conclusions:**

Understanding the roles of host, parasite, and vector rhythms on malaria transmission is important given that mosquitoes are altering the time of day that they bite. That rhythms, per se, do not affect vector-to-host transmission suggests that the impacts of time-of-day on components of vectorial capacity are more epidemiologically influential than host rhythms.

**Graphical abstract:**

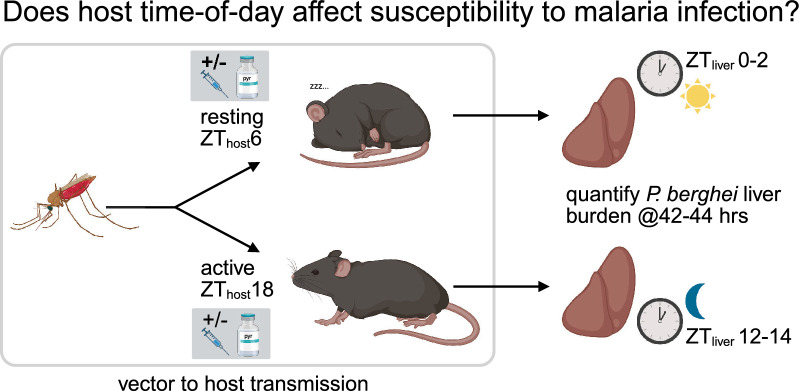

**Supplementary Information:**

The online version contains supplementary material available at 10.1186/s13071-025-06986-7.

## Background

Cyclical exposure to light and dark, resulting from the Earth’s daily rotation, exposes organisms to predictable daily variations in their environment. To optimise fitness by anticipating these daily environmental changes, most organisms use circadian clocks to govern behaviour, physiology and cellular processes. Disruptions to circadian rhythms are associated with many disorders. For example, long-term shift work increases the risk of metabolic disorders, hypertension, cardiovascular diseases and cancer [1]. In addition, circadian control of many aspects of the immune response [2] underpins the severity of disorders, illustrated by rheumatoid arthritis causing more intense joint pain in the morning [3]. Rhythms in immune responses and other host physiologies also affect host–pathogen interactions, including susceptibility to infection, pathogen survival and replication, and the severity of disease symptoms [4–6]. For example, circadian migration of immune cells modulates the availability of host cells that *Leishmania* parasites require to establish an infection in a mammalian host [7], and rhythms in host food intake influence the timing and the rate of asexual replication of malaria (*Plasmodium* spp.) parasites within red blood cells [8–10]. The constraints and opportunities that parasites experience as consequences of host rhythms can alter the course of infections. For instance, mice infected with diverse pathogens (including *Salmonella enterica* Typhimurium, *Streptococcus pneumonia* , *Chlamydia muridarum* or vesicular stomatitis virus) experience less severe symptoms when infected at the start of, or during, their active phase [3, 11–13].

For infectious agents acquired through foraging and social interactions around resource acquisition, hosts are at greater risk of infection during their active phase. To counter this, rhythmic immune defences are thought to be upregulated in the active phase, regardless of whether hosts are diurnal or nocturnal. Whether this is a general rule is unclear because some immune rhythms are likely to be due to homeostasis, not defence. For example, *Drosophila* infected in their rest phase (night) are more likely to survive infections of *Streptococcus pneumoniae* or *Pseudomonas aeruginosa* [14]; and herpes (MuHV-4 and HSV) viral loads, which are associated with pathogenicity, are lower in mice infected during (the latter part of) their rest phase [15, 16]. Nonetheless, that time-of-day of infection affects infection processes is well established across a wide range of host–pathogen combinations. Understanding why time-of-day dictates host susceptibility and pathogen performance is important given that many humans undertake shift work, and exposure to artificial light at night disrupts the daily rhythms of wildlife hosts and insect vectors [17]. Furthermore, for pathogens with complex, multi-host lifecycles, the time-of-day that pathogens encounter transmission opportunities varies. This is the case for malaria parasites whose mosquito vectors are shifting their blood foraging rhythm to bite earlier in the evening and later in the morning, likely to evade insecticide treated bed nets [18–21].

Resolving how disease severity and spread is affected by the time-of-day that malaria parasites transmit from host to vector, and from vector to host, is challenging because rhythms exhibited by host, parasite and vector all interact to determine the susceptibility of hosts and vectors, and the infectivity of parasites to each party. For example, during the night time, *P. chabaudi* transmission stages (gametocytes) are more infectious, but mosquitoes are less susceptible to malaria [22–25]. Interactions between rhythms of the host, parasite and vector are thought to influence transmission from vector to host [26], but the identity of these rhythms and their relative importance are unclear. Vertebrate hosts are infected with malaria when infected mosquitoes inject sporozoites into the skin during a blood meal. Sporozoites migrate to the liver where they undergo extensive – but almost clinically silent – replication before establishing the blood stage of infection. During this early stage of infection, parasites experience a rhythmic environment. Both sporozoites and liver stages are exposed to the host’s immune response [27], much of which is rhythmic [2]. In addition, liver stage parasites develop within the highly rhythmic environment of the hepatocyte, in which ~20% of gene expression is rhythmic, synchronised with the timing of host feeding/fasting [28, 29]. Following liver egress, parasites undergo rhythmic cycles of asexual replication in the host’s red blood cells (intraerythrocytic development cycle, IDC). The IDC is historically famous for causing rhythmic disease symptoms, and is scheduled by *P. chabaudi* to align to the host’s feeding/fasting rhythm, which is beneficial for within-host survival [8, 30]. Furthermore, the time-of-day that antimalarial drugs are taken can affect drug efficacy, especially for short-acting drugs or drugs targeting a subset of IDC stages [31], and the dormancy response that young IDC stages mount is one of the underlying causes of treatment failure [32–35]. The rhythmic IDC also fuels the production of transmission stages (gametocytes), which are more infectious at night in *P. chabaudi*, when *Anopheline* mosquitoes usually take a blood meal [22–25]. Thus, understanding how rhythms affect malaria infection could inform how to reduce disease symptoms, improve antimalarial efficacy, and reduce transmission.

We predicted that time-of-day affects how well parasites establish liver infections on the basis of observations that: (i) IDC stages align replication to host feeding-fasting rhythms; (ii) the hepatocyte environment also synchronises to feeding–fasting rhythms; (iii) pre-erythrocytic malaria parasites are sensitive to temperature, serum, bicarbonate and glucose metabolites, which are rhythmic; and (iv) rhythmic aspects of the immune response could affect sporozoites travelling to the liver [2, 9, 10, 27–29, 36]. By standardising the time-of-day for *P. berghei* parasites and *Anopheles stephensi* mosquitoes used to initiate experimental infections, we directly test the impact of murine host time-of-day on the parasites’ success in establishing liver stage infections. Because fitness consequences of environmental rhythms are often more apparent when organisms are under stress [37], we test for time-of-day effects in untreated mice and mice treated with the antimalarial drug pyrimethamine. We expect that any host time-of-day effects stemming from immune and/or feeding rhythms that limit parasite productivity will be exacerbated when parasite replication is also limited by pyrimethamine. As such, we expect reduced liver parasite densities in drug-treated mice, especially when infected during their active phase. Using drug treatment also makes our approach relevant to human malaria infections because parasites encounter hosts affected by residual long-acting drugs, and seasonal or intermittent preventive treatment programmes.

## Methods

We conducted three experiments to test whether the murine host’s time-of-day affects their susceptibility to *Plasmodium* infection. We compared the performance of *P. berghei* during the establishment of infections in untreated and pyrimethamine-treated mice, following vector-to-host transmission during the host’s active phase (night time) or rest phase (day time). Because pyrimethamine has a long half-life, fast uptake, does not require to be metabolised into active ingredients and drug elimination is a first order process [38], host rhythms should not affect drug efficacy directly. By ensuring that parasites in the ‘active phase’ and ‘rest phase’ infection groups were exposed to the same drug concentrations, we tested the impact of time-of-day of infection (ToI) on the parasites directly, or indirectly if ToI and drug treatment effects are not additive, but synergistic or antagonistic. To probe host rhythms in a manner unconfounded by parasite and/or vector rhythms, we used *P. berghei* that does not exhibit rhythmic replication in liver or IDC stages, nor experiences fitness costs when its IDC is misaligned to host rhythms [36, 39, 40], and we controlled for mosquito and parasite time-of-day at the point of infection (night time in all groups). We used pathogen density to approximate host susceptibility, avoiding the need for large sample sizes required to analyse binary (prevalence) compared with continuous (density) data.

### Hosts and vectors

Hosts were 6- to 8-week-old male C57/Bl6 mice housed at 21 °C in 12 h light:12 h dark photoschedules with ad libitum access to water and standard RM3 pelleted diet (SDS, UK). We entrained mice to their respective photoschedules (see below) for at least 2 weeks prior to infection, and throughout infections. We reared *An. stephensi* mosquitoes in a 12 h light:12 h dark photoschedule, at 28 °C and 60% relative humidity with access to 8% fructose post-emergence, supplemented with 0.05% para-aminobenzoic acid upon infection [41]. All procedures complied with UK Home Office regulations (Animals Scientific Procedures Act 1986; SI 2012/3039), the Animal Research: Reporting of In Vivo Experimental (ARRIVE) guidelines, and were approved following ethical review by the University of Edinburgh (PP8390310).

### Infections, drug treatment and sampling

Mosquitoes were infected with *P. berghei* following Spence et al. [41] with minor adjustments. Briefly, we allowed 24-h starved, 5–7-d old mosquitoes to blood feed for 15 min on donor mice (C57/Bl6 males), which were infected with *P. berghei* ANKA (Pb-WT-A2/28) 5 d previously. We anaesthetised donor mice prior to mosquito feeds by intraperitoneal injection of 500 µg/kg medetomidine and 50 mg/kg ketamine hydrochloride, and euthanised mice after the feed. Mosquitoes were kept at 21 °C from 2 d prior to the blood meal and throughout infections. On day 21 post blood meal, we administered pyrimethamine to naïve recipient mice by intraperitoneal injection at doses ranging from 0 (i.e. carrier only) to 5 mg/kg dissolved in 50 µL DMSO, 1 h before infection by mosquito bite so that maximum plasma concentrations coincided with transmission [38]. We allowed mosquitoes (*n* = 6/mouse) to feed on these naïve recipient mice, as described above, after which, anaesthetics were reversed by subcutaneous injection of 2.5 mg/kg atipamezole. Subsequently, we registered mosquito feeding status, bisected mosquitoes following [42] and froze the head/thorax section at −20 °C for DNA extraction and sporozoite quantification. To quantify parasite burdens in recipient mice, we euthanised them with pentobarbital injection at 42–44 h post infection (PI) and harvested their livers, which is before parasites egress to the blood, and froze livers at −80 °C until RNA extraction.

### Experimental designs

**Fig. 1:**
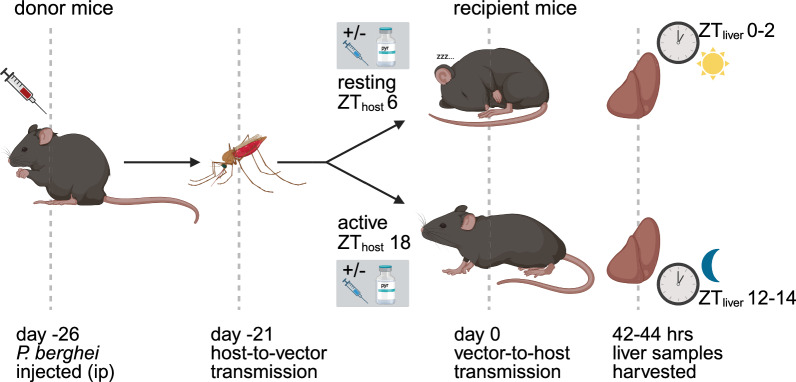
Experimental design. Donor mice were infected with *P. berghei* by intraperitoneal (ip) injection of IDC stages. On day 5 post-infection, mosquitoes were allowed to feed on gametocyte positive mice. Subsequently, 21 d later, recipient hosts were infected by mosquito bite during their active (night time, ZT_host_18) or rest phase (daytime, ZT_host_6). We administered either placebo or pyrimethamine to recipient hosts 1 h before vector-to-host transmission and collected liver samples 42–44 h after transmission to quantify the impact of host rhythms on parasite liver burdens. Times and clocks denote ZT times (time since lights on)

We tested whether hosts were more susceptible to *P. berghei* infection during their rest or active phase in untreated (0 mg/kg, carrier only) versus pyrimethamine-treated (2 mg/kg) hosts in experiment 1. We then tested whether the impacts of ToI are drug dose-dependent in our second (0, 1, 2 or 5 mg/kg pyrimethamine) and third (0.1, 0.2, 0.5 or 1 mg/kg pyrimethamine) experiments. Because we perturb the photoschedules of recipient mice relative to that of the parasites and mosquitoes used to initiate infections, ToI for each party is denoted as hours since the lights went on (Zeitgeber time, ZT) in their respective 12 h light:12 h dark photoschedules. For all experiments, we infected recipient mice by mosquito bite during the host active (night time, ZT_host_18) or rest phase (day time, ZT_host_6), using mosquitoes and parasites at ZT_mosq.para_18, reflecting naturally occurring nocturnal transmission times (Fig. 1). To account for potential effects of photoschedule manipulation on our results, our experimental design was achieved by either keeping mosquitoes (experiments 1, 2) or mice (experiment 3) in opposite photoschedules, as described below.

In experiments 1 and 2 (see Supplementary Fig. S1a, Additional File 1), we housed all donor and recipient mice in a light:dark (LD) photoschedule (lights on 07.00–19.00 GMT), whilst mosquitoes were kept either in LD or in the reversed “DL” photoschedule (lights on 19.00–07.00 GMT). We allowed pots of uninfected female LD mosquitoes (ZT_mosq_8 or 12 in experiments 1 and 2) and DL mosquitoes (ZT_mosq_20 or 24/0 in experiments 1 and 2) to simultaneously feed on a gametocyte-infected donor host (ZT_host_8, *n* = 4 or ZT_host_12, *n* = 5 for experiments 1 and 2) to generate infected mosquitoes (*n* = 60–80 mosquitoes/pot). This approach controls for potential donor effects and is expected to achieve similar transmission success in both groups [23]. Once parasites completed development within mosquitoes, we exposed 40 recipient mice to mosquito bites at 13.00 GMT using DL mosquitoes (ZT_host_6, ZT_mosq.para_18) or at 01.00 GMT using the LD mosquitoes (ZT_host_18, ZT_mosq.para_18), randomised from different donor mice across treatment groups. We administered pyrimethamine to recipient mice, 1 h before infection, at doses of 0 and 2 mg/kg (experiment 1; *n* = 10/ToI for each dose) or 0,1,2 and 5 mg/kg (experiment 2: *n* = 5/ToI for each dose). Livers were collected 42–44 h post-infection for all groups, i.e. 12 h apart for the ToI groups.

In experiment 3 (see Supplementary Fig. S1b, Additional File 1), we housed mice in either in LD (lights on 10.00–22.00 GMT) or in the reversed photoschedule DL (lights on 22.00–10.00 GMT). We kept all mosquitoes in the DL photoschedule so host-to-vector transmission, using pots of 60–80 mosquitoes/host, occurred at ZT_host_16 and ZT_mosq_16 using *P. berghei* gametocyte-infected hosts from the DL photoschedule (*n* = 5 donor mice). Recipient mice were infected at 16.00 GMT by allowing mosquitoes to bite LD hosts (ZT_host_6, ZT_mosq.para_18, *n* = 20) or DL hosts (ZT_host_18, ZT_mosq.para_18, *n* = 20). As for experiments 1 and 2, pots of mosquitoes that were infected from different donor mice, were randomised across recipient mice by treatment groups. Pyrimethamine was administered 1 h before infection at 0.1, 0.2, 0.5 or 1 mg/kg (*n* = 5/ToI for each dose).

### Parasite quantification

We extracted RNA from liver samples following [43] with minor modifications. Briefly, livers were homogenised in 5 mL TRIzol, from which we used 1 mL for RNA extraction and DNAse treatment [43]. Following further purification by isopropanol precipitation, we used 1 µg of RNA to produce cDNA in a 20µL volume (Thermofisher, 4368814), and 2 µL cDNA to quantify *P. berghei* liver parasite burdens by qPCR [43] (Table 1). Liver burdens are presented as *P. berghei* 18S gene expression relative to that of mouse β-actin, using the ΔΔCt method [44] to take into account variations in qPCR efficacy between the 18S and β-actin assays, and were calibrated using a *P. berghei* infected liver sample as a reference sample (samples A, B and C in experiments 1, 2 and 3, respectively).

**Table 1:** Quantitative PCRs to quantify parasite liver burden

Assay	Primer	Primer sequence
*P. berghei*	*Pb*liv.18S.fw	5′-AAGCATTAAATAAAGCGAATACATCCTTAC-3′
	*Pb*liv.18S.rv	5′-GGAGATTGGTTTTGACGTTTATGTG-3′
Mouse	mouseβ-actin.fw	5′- GGCTGTATTCCCCTCCATCG -3′
	mouseβ-actin.rv	5′- CCAGTTGGTAACAATGCCATGT -3′

Sequences of forward (fw) and reverse (rv) primers for parasite 18S and mouse β-actin quantitative PCRs used to quantify parasite liver burdens [43]. PCRs used 1× SYBR Green Master Mix (Thermofisher, 4309155), primers at 320 nM, and a program of 95 °C for 15 min followed by 40 cycles of 95 °C for 20 s, 60 °C for 30 s and 72 °C for 50 s. Amplification efficacy (± standard error, sem) for experiments 1, 2 and 3 was 90 ± 8%, 80 ± 1% and 86 ± 2% for *P. berghei* 18S, and 77 ± 2%, 76 ± 2% and 84 ± 1% for mouse β-actin, respectively. *P. berghei* PCR results correlate well with parasite numbers (*r*^2^ > 0.98) and no amplification of parasite 18S was detected in uninfected liver samples

We quantified sporozoites by extracting DNA from only the head–thorax sections, avoiding any parasites remaining in the abdomen from confounding counts ([23], modified from [45]). We quantified parasite genomes/mosquito by qPCR targeting the *Plasmodium* 18S rRNA gene following [46] (Table 2), using a quantification standard of microscopically quantified *P. berghei* IDC stages. The qPCR estimates the residual sporozoite load per mosquito, which is positively correlated both to the number of sporozoites expelled into the skin and to infection probability [47, 48]. Therefore, we summed the residual sporozoite load/mosquito over all mosquitoes that fed on a recipient host to approximate the total sporozoite load that mouse was exposed to (‘sporozoite load’ henceforth).

**Table 2:** Quantitative PCRs to quantify sporozoites

Assay	Primer/probe	Primer/probe sequence
*P. berghei*	*Pb*spor.18S.fw	5′-TGTCAGAGGTGAAATTCTTAGATTTTCT-3′
	*Pb*spor.18S.rv	5′-ACTTTCGTTCTTGATTAATGGAAGTATTT-3′
	*Pb*spor.18S.probe	5′-6-FAM-CAAACAACTGCGAAAGC-3′

Names and sequences for forward (fw) and reverse (rv) primers and Taqman probe (probe) for parasite 18S quantitative PCR used to quantify sporozoites [46]. The PCR used 1× TaqMan Fast Universal PCR mastermix (Thermofisher, 4352042) with 300 nM primers, 200 nM probe and a program of 95 °C for 20 min; 45 cycles of: 95 °C for 3 s, 60 °C for 30 s. Amplification efficacy (± sem) was 104 ± 3%, 92 ± 2% and 95 ± 2% for experiments 1, 2 and 3, respectively, and results correlated with the number of parasite genomes in the quantification standard (*r*^2^ > 0.99). We detected no amplification in uninfected mosquitoes

### Analyses

We analysed our data in R v4.5.0 [49]. We use linear models with a Gaussian error distribution to test for the impacts of ToI, drug dose (PYR) and their interaction upon sporozoite load and liver parasite burden. To analyse prevalence, we used generalised linear models with a binomial error structure. For all models, we used the DHARMa 0.4.7 package to check whether model assumptions of a.o. homogeneity of variance hold. Sporozoite loads for experiment 1 were log_10_ transformed. Liver parasite burden was analysed as ‘log_2_ relative expression’ and we controlled for variation in sporozoite load by including it as a fixed variable. Effect sizes for transformed variables are back transformed to illustrate fold-change differences. We used an information-theoretic approach [50], which does not result in accepting/rejecting hypotheses based on *P*-values as in traditional null hypothesis testing, but takes into account the uncertainty of alternative statistical models (i.e. hypotheses), and quantifies the relative support for a predetermined set of hypotheses. We used the MuMIn v1.47.5 package, and present the models most likely to explain the variation in our data based on AICc (Akaike’s information criterion corrected for small sample bias) (ΔAICc < 2) with their relative weights (AICc weight), and the proportion of variance explained (*r*^2^). The AICc weights reflect the uncertainty of the model’s contribution to explaining the variation in the data. We estimate mean effect sizes ± sem across competitive models (ΔAICc < 2) by using a weighted average across all competitive models in which the parameter is present (i.e. the conditional average). Graphs present means ± sem from the data and, for ease of interpretation, we translate effect sizes into fold-change, or present predicted effect sizes when sporozoite load requires accounting for (i.e. for liver parasite burden). In Additional File 1, we present Tables S1–S3 with all tested models.

## Results

### Experiment 1

**Fig. 2:**
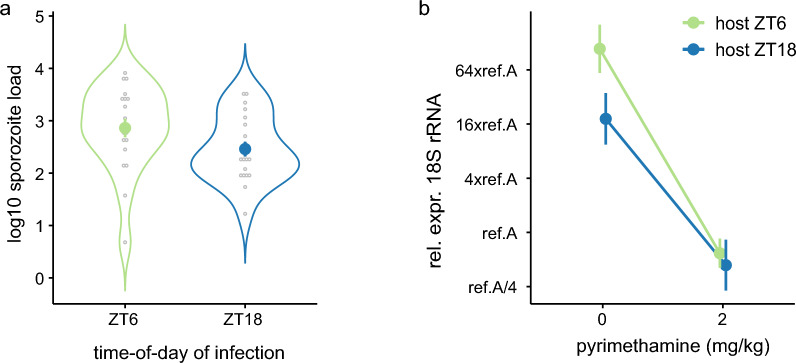
Sporozoite load and liver parasite burden for infected hosts in experiment 1. Mean (± sem) sporozoite load (**a**) and liver parasite burden for infected hosts (**b**) for hosts infected during their active (ZT_host_18) or rest (ZT_host_6) phase. Liver burden for placebo- and pyrimethamine (2 mg/kg)-treated hosts in **b** is the RNA expression of parasite 18S, adjusted for mouse *GAPDH*, and relative to the *P. berghei* reference sample A (ref.A)

We tested whether the time-of-day of infection (ToI, ZT_host_6 or 18) affects *P. berghei* liver burden in untreated and pyrimethamine-treated hosts (2 mg/kg). Sporozoite loads did not differ between pyrimethamine groups (Supplementary Table S1a), but were different between ToI groups (ΔAICc = 0, AICc weight = 0.43, *r*^2^ = 0.08) with ZT_host_18 mice receiving 2.51-fold (log_10_ spor −0.40 ± sem 0.23) less sporozoites than those infected at ZT_host_6 (Fig. 2a). However, confidence in this difference is low because the null model was also competitive (ΔAICc = 0.80, AICc weight = 0.29, *r*^2^ = 0). Nonetheless, to be conservative, we included sporozoite load in subsequent analyses to account for any impact it may have on liver burden.

We detected parasites in the liver for 75% of mice across all treatment groups, similar to previous transmission studies [47], with 15/20 patent mice in both ToI groups (ZT_host_18: 9/10 untreated and 6/10 drug-treated; ZT_host_6: 7/10 untreated and 8/10 drug-treated). We find no evidence that prevalence varied between ToI groups (Supplementary Table S1b), and whilst the model with pyrimethamine only (ΔAICc = 1.68, AICc weight = 0.21, *r*^2^ = 0.01) was considered competitive with the null model (ΔAICc = 0, AICc weight = 0.50, *r*^2^ = 0), it explains only 1% of the variance with high model uncertainty, so we conclude that untreated and treated-mice were equally likely to be infected.

Liver burdens of patent mice are best explained by variations in pyrimethamine treatment (ΔAICc = 0, AICc weight = 0.46, *r*^2^ = 0.72), with weaker support for the impact of ToI (ΔAICc = 1.38, AICc weight = 0.23, *r*^2^ = 0.74) and ToI-dependent modulation of drug efficacy (ToI by pyrimethamine interaction: ΔAICc = 0.74, AICc weight = 0.32, *r*^2^ = 0.77) (Fig. 2b; Supplementary Table S1c). Overall, accounting for variations in sporozoite loads, hosts exhibited 3.2-fold lower liver burdens when infected during their active compared with their rest phase in untreated infections, whilst no ToI difference was observed in drug-treated infections (effect sizes ± sem for ToI: −1.70 ± 1.15; ToI by pyrimethamine interaction: 2.85 ± 1.55) (Fig. 2b).

### Experiment 2

**Fig. 3:**
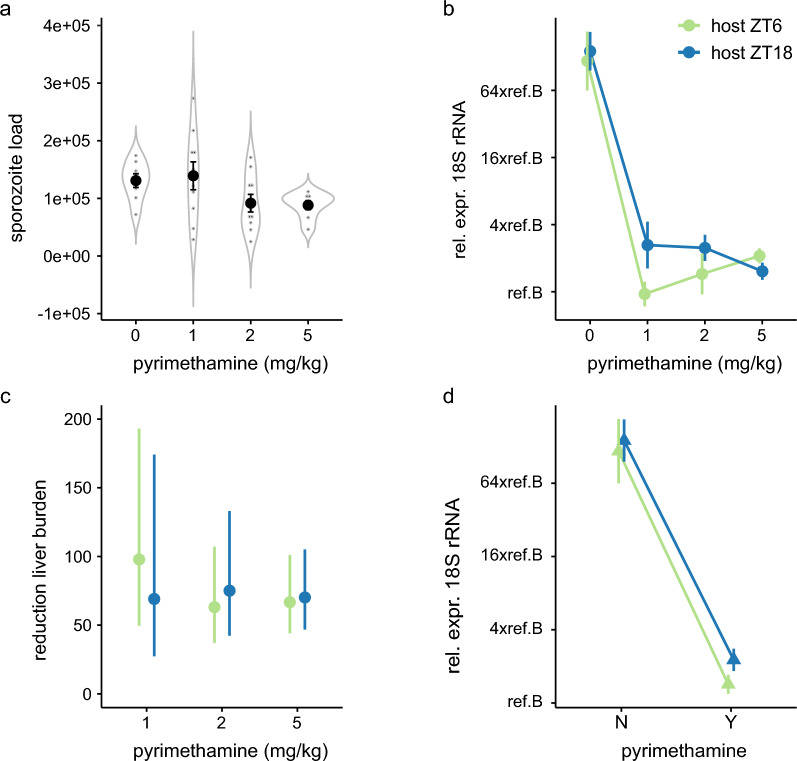
Sporozoite load, liver parasite burden and drug efficacy for hosts in experiment 2. Mean (± sem) sporozoite load (**a**), liver parasite burden (**b**,**d**) and pyrimethamine efficacy (**c**) for hosts treated with pyrimethamine at doses of 1, 2 and 5 mg/kg or placebo. Liver parasite burdens (**b**, **d**) for hosts infected during their active (ZT_host_18) or rest phase (ZT_host_6), are presented as RNA expression of parasite 18S, adjusted for mouse *GAPDH*, and relative to *P. berghei* reference sample B (ref.B) and grouped by pyrimethamine dose (mg/kg, **b**) or whether hosts received pyrimethamine (Y) or not (N) (**d**). Pyrimethamine dose–response (**c**) is presented as the *x*-fold reduction in liver burden for hosts infected during their active (ZT_host_18) or rest phase (ZT_host_6), relative to their respective untreated groups

Having observed weak support for parasites performing better following infection during the rest phase of untreated hosts, we investigated a dose–response by extending the range of doses tested to 0, 1, 2 or 5 mg/kg pyrimethamine. Of 40 exposed mice (5/ToI for each dose), data from 4 could not be analysed due to failed liver RNA extractions (ZT_host_6: 0 mg/kg *n* = 1; ZT_host_18: 0 mg/kg *n* = 1 and 5 mg/kg *n* = 2). Unlike for experiment 1, sporozoite loads did not vary between ToI groups (Supplementary Table S2a), but did vary between pyrimethamine groups (pyrimethamine: ΔAICc = 0.40, AICc weight = 0.35, *r*^2^ = 0.18) (Fig. 3a). Overall, sporozoite loads were 0.94, 1.43 and 1.48-fold lower in groups receiving 1, 2 or 5 mg/kg pyrimethamine, compared with untreated mice, which received on average 1.3 ± 0.1 × 10^5^ sporozoites (effect sizes ± sem relative to untreated mice 0.08 ± 0.25 × 10^5^; −0.39 ± 0.25 × 10^5^; and −0.43 ± 0.27 × 10^5^ for 1, 2 and 5 mg/kg, respectively). Again, confidence in this difference is low because the null model is also competitive (ΔAICc = 0, AICc weight = 0.43, *r*^2^ = 0). As for experiment 1, we included sporozoite load in subsequent analyses to account for any impact it may have on liver burden.

*P. berghei* liver stages were detected in all 36 mice, and liver burden is explained by additive effects of ToI and pyrimethamine dose (ΔAICc = 0, AICc weight = 0.63, *r*^2^ = 0.89) but not their interaction (Fig. 3b; Supplementary Table S2b). There is less confidence in the impact of ToI on liver burden because the model including pyrimethamine only was also competitive (ΔAICc 1.16, AICc weight = 0.35, *r*^2^ = 0.88). We did not observe a dose–response for pyrimethamine treatment on liver burdens (Fig. 3c) and so compared mice who did (1, 2 and 5 mg/kg combined) or did not (0 mg/kg) receive pyrimethamine (Fig. 3d). This analysis also reveals additive effects of ToI and pyrimethamine dose, with weaker support for ToI (pyrimethamine and ToI: ΔAICc = 0, AICc weight = 0.50, *r*^2^ = 0.88; PYR: ΔAICc = 0.61, AICc weight = 0.37, *r*^2^ = 0.87; Supplementary Table S2c). Having accounted for sporozoite loads, drug treatment per se reduced liver burden 63.3-fold relative to untreated infections (effect size PYR: −5.98 ± sem 0.44). With much weaker support, mice infected during their active phase (ZT_host_18) experienced 1.5-fold higher liver burdens (effect size ToI: 0.61 ± sem 0.36) regardless of pyrimethamine dose (Fig. 3d).

### Experiment 3

**Fig. 4:**
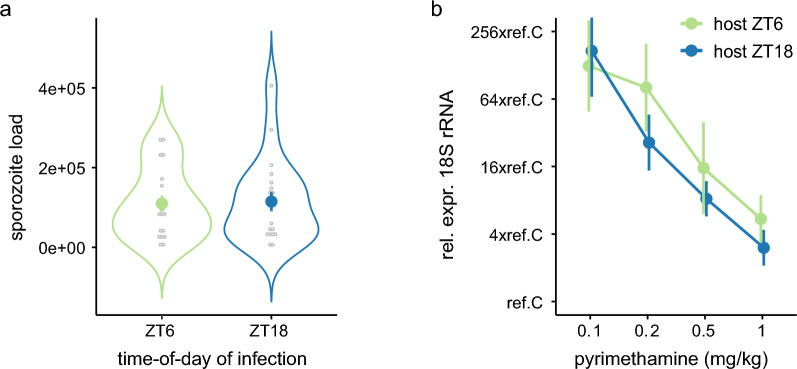
Sporozoite load and liver parasite burden for hosts in experiment 3. Mean (± sem) sporozoite load (**a**) and liver parasite burden (**b**) for hosts infected during their active (ZT_host_18) or rest phase (ZT_host_6). Liver burdens for pyrimethamine (0.1, 0.2, 0.5 or 1 mg/kg) treated hosts in **b** are RNA expression of parasite 18S, adjusted for mouse *GAPDH*, and relative to *P. berghei* reference sample C (ref.C).

To test whether the lack of dose response in experiment 2 occurred because the upper limit of drug efficacy was reached at 1 mg/kg pyrimethamine, we examined a lower range of doses (0.1, 0.2, 0.5 or 1 mg/kg). We did not include untreated infections because our aim was to test whether ToI modulates the dose–response in liver burdens. Of the 40 exposed mice (*n* = 5/ ToI for each dose), we excluded 2 (ZT_host_6: 0.5 mg/kg *n* = 1, ZT_host_18: 0.1 mg/kg *n* = 1) mice from all analyses because RNA samples failed. In contrast to experiments 1 and 2, sporozoite loads did not differ between any groups, with the null model being the only competitive model (ΔAICc = 0, AICc weight = 0.74, *r*^2^ = 0) (Fig. 4a; Supplementary Table S3a). On average, each mouse was exposed to 1.12 ± sem 0.15 × 10^5^ sporozoites. Nonetheless, for consistency, sporozoite load is accounted for in all liver burden analyses.

Liver burdens were patent in all 38 mice and we find strong support that pyrimethamine affects liver burdens (ΔAICc = 0, AICc weight = 0.65, *r*^2^ = 0.57), and weaker support for pyrimethamine and ToI having additive (ΔAICc = 1.26, AICc weight = 0.35, *r*^2^ = 0.59) but not synergistic effects (Fig. 4b; Supplementary Table S3b). Drug treatment reduced liver burden in a dose-dependent manner at both ToI. Relative to 0.1 mg/kg, the higher doses reduced liver burdens 3.2-fold (0.2 mg/kg), 13.8-fold (0.5 mg/kg) and 36.4-fold (1 mg/kg) (effect sizes ± sem relative to 0.1 mg/kg: −2.00 ± 0.96; −4.00 ± 0.99; and −5.32 ± 0.96, respectively). With weaker support, mice infected during the active phase (night time, ZT_host_18) had 1.9-fold lower liver burdens across all drug doses than mice infected during their rest phase (effect size −0.80 ± sem 0.67).

## Discussion

Following observations that susceptibility to many infectious diseases is time-of-day dependent and often lower in the host’s active phase [3, 11–16, 22–25], we asked if this also occurs when malaria parasites infect the vertebrate host. We tested for the overall impact of a large number of host rhythms, while controlling for mosquito and parasite rhythms. We performed our experiments in untreated and drug-treated hosts because impacts of host rhythms may be more apparent in stressed parasites. As expected, pyrimethamine treatment consistently reduced liver burdens, but drug treatment did not eliminate liver parasites even at the highest doses used. We found no strong evidence that host time-of-day (active/rest phase) impacts susceptibility to *P. berghei* in a consistent manner. Mice infected during their active phase harbour 3.2-fold *fewer* parasites (but only for untreated mice) in experiment 1, 1.5-fold *more* parasites in experiment 2, and 1.9-fold *fewer* in experiment 3. Moreover, because statistical confidence in the impact of ToI is low, and the direction of the ToI effect sizes is inconsistent between experiments, we conclude that ToI does not affect parasite liver burdens in a biologically significant manner.

That time-of-day does not clearly impact *P. berghei* productivity during the establishment of liver infections was unexpected. A statistically significant impact of host ToI on susceptibility to *P. berghei* was recently reported, although with a similarly small effect size [26]. While our experiments differed from [26] because we used mosquito bites as opposed to injected sporozoites, all three of our experiments gave contrasting results. Our experiments also included different designs (i.e. whether experimental hosts were housed in the same or different photoschedules), drug doses and overall sporozoite loads, but these factors are unlikely to have generated contradictory findings for the following reasons. First, experiments 1 and 2 used the same experimental design, yet yielded opposite ToI effects. Second, sporozoite loads were considerably higher in experiment 1 than experiments 2 and 3, but opposite ToI results arose from experiments 2 and 3, and moreover, variation in sporozoite load was statistically controlled for. Third, age differences can affect sporozoite gene expression, motility and immunogenicity, and hosts in experiments 1 and 2 were infected using sporozoites whose age differed by 12 h at each ToI. Yet, liver burdens were not consistently higher for infections initiated with older, more mature sporozoites. Alternatively, the generally high sporozoite inoculum of the *P. berghei* model may cause high parasite loads to overshadow time-of-day effects. However, if this were the case, it would mean that time-of-day effects are relatively small and likely biologically irrelevant. Fourth, our data cannot be explained by between-host variation either, because all experiments used age-matched male C57/Bl6 mice. A final possibility is that by collecting data at two time points, there is a risk of ‘accidentally’ detecting the same trait value when probing a symmetrical rhythm 12 h apart (i.e. as the symmetrical curve ascends and descends). However, by sampling in the middle of the rest and active phase, when trait values for both immune rhythms and nutrient levels are considerably different [51], our design avoids that issue. Therefore, we conclude that the absence of a consistent directional difference in liver burdens for hosts infected during their active versus rest phase is not a product of our approaches.

Intuitively, one might expect that, because the fitness of asynchronously replicating blood stage *P. berghei* is unaffected by acute misalignment with host rhythms [39, 40], this could extend to other parts of its life cycle. However, *P. berghei* exhibits rhythmic gene expression in the vector and the fitness of other non-rhythmically replicating pathogens are mediated by host rhythms in the expression of receptors for cell entry, metabolism and immune factors [13, 15, 16, 26, 52]. The absence of a ToI effect upon *P. berghei* liver stages might be due to immune rhythms and nutrient rhythms having equal but opposing effects, thus cancelling out any overall impact upon the parasites. Transmission during the host rest phase may be beneficial for sporozoites because fewer are killed by the immune system, but also means that the first 12 h of liver schizogony (18–30 h later) occur during the next rest (i.e. fasting) phase when low nutrients may limit parasite replication. To disentangle such opposing effects, further experiments could alter the relative impacts of immune rhythms versus nutrient rhythms. This could include manipulating the timing of host immune rhythms and/or feeding rhythms (using time restricted feeding [30] and conditional clock knock outs of immune cells), investigating liver burdens at an earlier time point (before feeding rhythms have substantial impact) and testing sporozoite motility in skin models. When synchronised, primary human hepatocytes in vitro exhibit time-of-day dependent susceptibility to sporozoite infection in the absence of immune cells [26, 53]. However, translating these approaches to conduct ecologically realistic studies is a huge challenge. Malaria parasites have evolved a suite of strategies to avoid immune attack, including cell traversal, remodelling of host cells, rosetting, sequestration and variable antigen expression, and can manipulate host cells to obtain nutrients [54, 55]. Parasites may have evolved to evade rhythmic immune responses that target sporozoites, and have foraging strategies to ensure they are not nutrient-limited within hepatocytes, enabling non-rhythmic, extensive replication within hepatocytes [36]. Therefore, the impact of time-of-day may only manifest when parasites enter the blood stage of infection where they face stronger pressures from immune responses and nutrient limitation [56].

Given reports that mosquitoes are shifting their biting times to evade the widespread use of bednets [18–21], understanding the impact of daily rhythms on malaria transmission in untreated and drug-treated hosts is timely and important. We designed our drug treatments to minimise potential influences of host rhythms (e.g. drug absorption or drug clearance) on the bioavailability of drugs. However, such rhythmic impacts could occur for prodrugs that require metabolic activation, in which case time-of-day of administration of prophylaxis may be important [55]. However, if the results of our experiments – that host rhythms per se have a minor, if any, impact on vector to host transmission – apply to human parasites, this suggests that previously demonstrated time-of-day effects on transmission from host to vector [22–25] are more epidemiologically influential than host time-of-day at infection. Even if host rhythms do not affect parasite productivity in the early phase of malaria infection, it is likely that the rhythms of vectors and incoming sporozoites do matter [22–26, 57–59]. For example, mosquitoes may take larger blood meals during their active phase [26] (but see [24, 60]) and exhibit daily rhythms in salivary gland transcripts and proteins associated with blood-feeding [26]. As mosquito saliva can influence sporozoite motility and host responses to mosquito bites [26, 58, 59], such rhythms may affect transmission. In addition, heterogeneity and daily rhythms in sporozoite gene expression may affect their motility and immunogenicity following inoculation into the skin [26, 61]. These additive or interactive contributions of rhythms may explain why liver burdens are approximately twofold higher in mice infected during their active versus rest phase with sporozoites inoculated from night time mosquitoes, but not when sporozoites originate from daytime mosquitoes [26].

## Conclusions

We tested whether vertebrate hosts, like mosquito vectors, are less susceptible to malaria during their active versus rest phase. We conclude that host time-of-day does not have a biologically relevant impact on vector-to-host transmission in either pyrimethamine-treated or untreated hosts. If our results apply to human parasites, daily rhythms in components of vectorial capacity are more epidemiologically influential than host rhythms, which is important because mosquitoes are altering the time-of-day they bite.

## Supplementary Information

Additional file 1. Figure S1. Experimental design, achieved by either keeping mosquitoes (a, experiments 1 and 2) or mice (b, experiment 3) in opposite photoschedules. Table S1. Relative support for each tested linear or generalised linear model in explaining the impact of time-of-day of infection, drug treatment and their interactions on sporozoite load, infection prevalence and parasite liver burden, for experiment 1. Table S2. Relative support for each tested linear or generalised linear model in explaining the impact of time-of-day of infection, drug treatment and their interactions on sporozoite load and parasite liver burden, for experiment 2. Table S3. Relative support for each tested linear or generalised linear model in explaining the impact of time-of-day of infection, drug treatment and their interactions on sporozoite load and parasite liver burden, for experiment 3.

## Data Availability

The data supporting the findings in this study are deposited in the Edinburgh Datashare at https://doi.org/10.7488/ds/7981 . The rodent malaria line *P. berghei* ANKA can be obtained from the European Malaria Reagent Repository (http://www.malariaresearch.eu).

## Abbreviations

IDC: Intraerythrocytic development cycle
ToI: Time-of-day of infection
PYR: Pyrimethamine
ZT: Zeitgeber time
LD: Light:dark photoschedule
DL: Reversed photoschedule
GMT: Greenwich mean time
AICc: Akaike’s information criterion corrected for small sample bias
