# Supplementary material for: Time-of-day of infection: impact on liver stage malaria parasites in untreated and drug-treated hosts
Source: Parasit Vectors. 2025 Aug 6;18:339. doi: 10.1186/s13071-025-06986-7 (PMC12329948; doi:10.1186/s13071-025-06986-7)
Supplement: Supplementary file 1 — Additional file 1. Figure S1. Experimental design, achieved by either keeping mosquitoes (a, experiments 1 and 2) or mice (b, experiment 3) in opposite photoschedules. Table S1. Relative support for each tested linear or generalised linear model in explaining the impact of time-of-day of infection, drug treatment and their interactions on sporozoite load, infection prevalence and parasite liver burden, for experiment 1. Table S2. Relative support for each tested linear or generalised linear model in explaining the impact of time-of-day of infection, drug treatment and their interactions on sporozoite load and parasite liver burden, for experiment 2. Table S3. Relative support for each tested linear or generalised linear model in explaining the impact of time-of-day of infection, drug treatment and their interactions on sporozoite load and parasite liver burden, for experiment 3. [file 13071_2025_6986_MOESM1_ESM.pdf]

Additional File 1

Time-of-day of infection: impact on liver stage malaria parasites in untreated and drug treated hosts

Petra Schneider[1*], Aidan J. O'Donnell[1], Alejandra Herbert-Mainero[1,2] and Sarah E. Reece[1]

[1] Institute of Ecology and Evolution, University of Edinburgh, Edinburgh, UK.

[2] Department of Human Genetics, University of Utah, Salt Lake City, USA.

[*]Corresponding author: Petra Schneider, petra.schneider@ed.ac.uk

Supplementary Figure S1 describes the experimental design, achieved by either keeping mosquitoes (**a**, experiments 1 and 2**)** or mice (**b**, experiment 3) in opposite photoschedules. **Supplementary Tables S1-S3** present the relative support for each tested linear model (lm) or generalised linear model (glm, using a binomial error structure to analyse prevalence). Presented are the intercept and parameters, including host time-of-day of infection (ToI), drug treatment (PYR) and their interaction (ToI * PYR). Sporozoite load (spor) is a fixed parameter in all liver burden models, to correct for between-host variation in exposure. Liver parasite burdens are analysed as by $\log_2$ relative gene expression (*P. berghei* 18S relative to mouse β-actin gene expression). For each model the proportion of variance explained (r^2), degrees of freedom (df), log likelihood (logLik) and Akaike's Information Criterion, corrected for small sample bias (AICc) is shown. ΔAICc and the models' relative AICc weights (weight) reflect the uncertainty of the model's contribution to explaining the variation in the data.

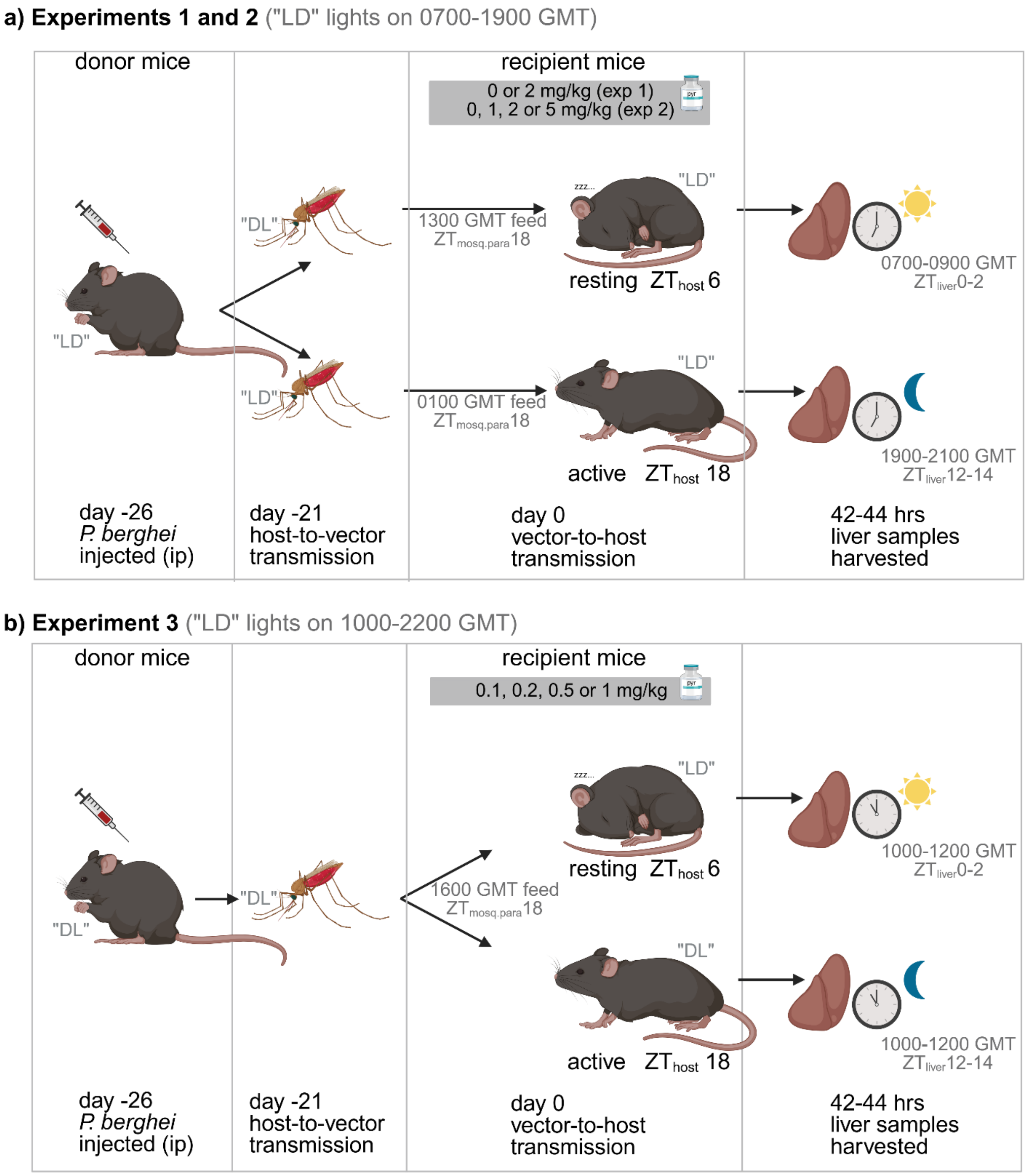

Figure S1. Experimental design, achieved by either keeping mosquitoes (**a)** or mice (**b**) in opposite photoschedules. (**a**) In experiments 1 and 2, mice were housed in an LD photoschedule (light 0700-1900 GMT), whilst mosquitoes were either in “LD” or in the reversed “DL” photoschedule (light 1900-0700 GMT). Donor mice were infected with *P. berghei* IDC stages by intraperitoneal (ip) injection. On day 5 post infection, we allowed pots

of uninfected female LD ($ZT_{mosq}8$ or 12 in experiments 1 and 2) and DL mosquitoes ($ZT_{mosq}20$ or 24/0 in experiments 1 and 2) to simultaneously feed on a gametocyte-infected donor host ($ZT_{host}8$, n=4; $ZT_{host}12$, n=5 for experiments 1 and 2), generating infected mosquitoes (n=60-80/pot). This approach is expected to achieve similar transmission success in both groups while controlling for potential donor effects. Twenty-one days later, recipient hosts were exposed to mosquito bite, during their active (night time, $ZT_{host}18$) or rest phase (daytime, $ZT_{host}6$). We achieved this by exposing mice to mosquito bites at 1300 GMT using DL mosquitoes ($ZT_{host}6$, $ZT_{mosq.para}18$) or at 0100 GMT using LD mosquitoes ($ZT_{host}18$, $ZT_{mosq.para}18$). We administered pyrimethamine to recipient mice, one hour before vector-to-host transmission, at doses of 0 and 2 mg/kg (experiment 1; n=10/ToI for each dose) or 0,1,2 and 5 mg/kg (experiment 2: n=5/ToI for each dose). Livers were collected 42-44 hrs post infection, i.e. 12 hours apart for the ToI groups. (**b**) In experiment 3, we housed mice in either in "LD" (light 1000-2200 GMT) or in "DL" (light 2200-1000 GMT). We kept all mosquitoes in the "DL" photoschedule. Host-to-vector transmission, using pots of 60-80 mosquitoes/host, occurred at $ZT_{host}16$ and $ZT_{mosq}16$ using *P. berghei* gametocyte-infected donors ("DL", n=5). Recipient mice were infected at 1600 GMT by allowing mosquitoes to bite LD hosts ($ZT_{host}6$, $ZT_{mosq.para}18$, n=20) or DL hosts ($ZT_{host}18$, $ZT_{mosq.para}18$, n=20). Pyrimethamine was administered one hour before infection at 0.1, 0.2, 0.5 or 1 mg/kg (n=5/ToI for each dose), and livers were collected 42-44 hrs post infection. For all experiments, pots of mosquitoes that were infected from different donor mice, were randomised across treatment groups. Times are given as ZT (time since lights on) or GMT (Greenwich mean time) and clocks denote GMT.

Table S1: Model selection tables experiment 1

a. Sporozoite load per mouse: lm(log_{10}(spor)~ToI*PYR)

Intercept	ToI	PYR	ToI*PYR	r^2	df	logLik	AICc	ΔAICc	weight
**2.861**	**+**	**-**	**-**	**0.0755**	**3**	**-42.65**	**92.0**	**0.00**	**0.430**
**2.660**	**-**	**-**	**-**	**0.0000**	**2**	**-44.22**	**92.8**	**0.80**	**0.288**
2.868	+	+	-	0.0756	4	-42.65	94.4	2.47	0.125
2.667	-	+	-	0.0001	3	-44.22	95.1	3.14	0.090
2.738	+	+	+	0.1073	5	-41.95	95.7	3.70	0.068

b. Infection prevalence per mouse: glm(prevalence~ToI*PYR)

Intercept	ToI	PYR	ToI*PYR	r^2	df	logLik	AICc	ΔAICc	weight
**1.0990**	**-**	**-**	**-**	**0.0000**	**1**	**-22.493**	**47.1**	**0.00**	**0.496**
**1.3860**	**-**	**+**	**-**	**0.0133**	**2**	**-22.225**	**48.8**	**1.68**	**0.214**
1.0990	+	-	-	0.0000	2	-22.493	49.3	2.22	0.164
1.3860	+	+	-	0.0133	3	-22.225	51.1	4.03	0.066
0.8473	+	+	+	0.0676	4	-21.094	51.3	4.24	0.060

c. parasite burden in the liver: lm(liver burden~ToI*PYR+spor)

Intercept	ToI	PYR	ToI*PYR	r^2	df	logLik	AICc	ΔAICc	weight
**4.530**	**-**	**+**	**-**	**0.7225**	**4**	**-64.376**	**138.4**	**0.00**	**0.456**
**5.800**	**+**	**+**	**+**	**0.7676**	**6**	**-61.719**	**139.1**	**0.74**	**0.316**
**5.178**	**+**	**+**	**-**	**0.7362**	**5**	**-63.619**	**139.7**	**1.38**	**0.228**
1.817	-	-	-	0.0325	3	-83.112	173.1	34.79	0.000
2.008	+	-	-	0.0339	4	-83.091	175.8	37.43	0.000

Lm: linear model; glm: generalised linear model; ToI: time-of-day of infection, PYR: pyrimethamine drug treatment; ToI*PYR: interaction between ToI and PYR; spor: sporozoite load; df: degrees of freedom, logLik: log likelihood; AICc: Akaike's Information Criterion corrected for small sample bias

Table S2: Model selection tables experiment 2

a. Sporozoite load per mouse: lm(spor~ToI*PYR)

Intercept	ToI	PYR	ToI*PYR	r^2	df	logLik	AICc	ΔAICc	weight
**112900**	**-**	**-**	**-**	**0.0000**	**2**	**-443.20**	**890.8**	**0.00**	**0.430**
**130900**	**-**	**+**	**-**	**0.1820**	**5**	**-439.58**	**891.2**	**0.40**	**0.351**
113800	+	-	-	0.0003	3	-443.19	893.1	2.37	0.131
133300	+	+	-	0.1839	6	-439.54	894.0	3.22	0.086
135700	+	+	+	0.2398	9	-438.26	901.4	10.69	0.002

b. parasite burden in the liver: lm(liver burden~ToI*PYR+spor)

Intercept	ToI	PYR	ToI*PYR	r^2	df	logLik	AICc	ΔAICc	weight
**5.170**	**+**	**+**	**-**	**0.8943**	**7**	**-47.850**	**113.7**	**0.00**	**0.631**
**5.540**	**-**	**+**	**-**	**0.8810**	**6**	**-49.980**	**114.9**	**1.16**	**0.353**
5.345	+	+	+	0.9040	10	-46.116	121.0	7.33	0.016
0.264	-	-	-	0.1073	3	-86.248	179.2	65.55	0.000
-0.114	+	-	-	0.1260	4	-85.867	181.0	67.33	0.000

c. parasite burden in the liver: lm(liver burden~ToI*PYR(Y/N)+spor)

Intercept	ToI	PYR	ToI*PYR	r^2	df	logLik	AICc	ΔAICc	weight
5.5490	+	+	-	**0.8802**	**5**	**-50.094**	**112.2**	**0.00**	**0.503**
5.8700	-	+	-	**0.8687**	**4**	**-51.754**	**112.8**	**0.61**	**0.371**
5.6690	+	+	+	0.8807	6	-50.023	114.9	2.75	0.127
0.2644	-	-	-	0.1073	3	-86.248	179.2	67.06	0.000
-0.1142	+	-	-	0.1260	4	-85.867	181.0	68.84	0.000

Lm: linear model; ToI: time-of-day of infection, PYR: pyrimethamine drug treatment; ToI*PYR: interaction between ToI and PYR; spor: sporozoite load; df: degrees of freedom, logLik: log likelihood; AICc: Akaike's Information Criterion corrected for small sample bias

Table S3: Model selection tables experiment 3

a. Sporozoite load per mouse: lm(spor~ToI*PYR)

Intercept	ToI	PYR	ToI*PYR	r^2	df	logLik	AICc	ΔAICc	weight
**112400**	**-**	**-**	**-**	**0.0000**	**2**	-489.18	982.7	**0.00**	**0.737**
109800	+	-	-	0.0007	3	-489.17	985.0	2.34	0.229
89180	-	+	-	0.0246	5	-488.71	989.3	6.59	0.027
87660	+	+	-	0.0249	6	-488.70	992.1	9.41	0.007
80810	+	+	+	0.0469	9	-488.27	1001.0	18.26	0.000

b. parasite burden in the liver: lm(liver burden~ToI*PYR+spor)

Intercept	ToI	PYR	ToI*PYR	r^2	df	logLik	AICc	ΔAICc	weight
**6.357**	**-**	**+**	**-**	**0.5683**	**6**	**-77.622**	**170.0**	**0.00**	**0.649**
**6.705**	**+**	**+**	**-**	**0.5879**	**7**	**-76.738**	**171.2**	**1.26**	**0.346**
6.217	+	+	+	0.6059	10	-75.892	179.9	9.98	0.004
3.626	-	-	-	0.0670	3	-92.265	191.2	21.28	0.000
4.109	+	-	-	0.0979	4	-91.625	192.5	22.51	0.000

Lm: linear model; ToI: time-of-day of infection, PYR: pyrimethamine drug treatment; ToI*PYR: interaction between ToI and PYR; spor: sporozoite load; df: degrees of freedom, logLik: log likelihood; AICc: Akaike's Information Criterion corrected for small sample bias
